# From Non-Alcoholic Fatty Liver to Hepatocellular Carcinoma: A Story of (Mal)Adapted Mitochondria

**DOI:** 10.3390/biology12040595

**Published:** 2023-04-14

**Authors:** Ricardo Amorim, Carina C. Magalhães, Fernanda Borges, Paulo J. Oliveira, José Teixeira

**Affiliations:** 1 CNC—Center for Neuroscience and Cell Biology, CIBB—Centre for Innovative Biomedicine and Biotechnology, University of Coimbra, 3004-504 Coimbra, Portugal; 2 CIQUP-IMS/Department of Chemistry and Biochemistry, Faculty of Sciences, University of Porto, 4169-007 Porto, Portugal

**Keywords:** non-alcoholic fatty liver disease (NAFLD), mitochondrial hepatic populations, mitochondrial adaptation, mitochondrial dysfunction

## Abstract

**Simple Summary:**

Non-alcoholic fatty liver disease (NAFLD) is a global pandemic that affects 25% of the world’s population and represents a serious health and economic concern worldwide resulting from unhealthy dietary habits combined with a sedentary lifestyle, although genetic contributions have been documented. Although the molecular mechanisms that cause the progression are not fully understood, metabolic-dysfunction-associated fatty liver disease is strong evidence that mitochondrial dysfunction plays a significant role in NAFLD. This review postulates that the regulation of hepatocytes’ mitochondrial physiology to maintain hepatic mitochondrial mass, integrity, and function are differently altered during NAFLD progression. This review summarizes evidence linking mitochondrial (dys)function with NAFLD pathophysiology, discriminating it in different disease stages (simple steatosis, steatohepatitis, liver fibrosis, cirrhosis, and hepatocellular carcinoma). As mitochondrial dysfunction is considered a driving force in NAFLD progression, targeting hepatocytes’ mitochondrial physiology could contribute to establishing an effective therapy for NAFLD. However, additional studies on distinct mitochondrial sub-populations roles in NAFLD, the impact of mitochondrial (mis)communication with other subcellular organelles (peroxisomes and lipid droplets), the impact of negligible pathways, such as fatty acid oxidation, de novo lipogenesis, and the pentose phosphate pathway in the hepatocytes’ mitochondrial physiology in different stages of NAFLD are topics to explore.

**Abstract:**

Non-alcoholic fatty liver disease (NAFLD) is a global pandemic affecting 25% of the world’s population and is a serious health and economic concern worldwide. NAFLD is mainly the result of unhealthy dietary habits combined with sedentary lifestyle, although some genetic contributions to NAFLD have been documented. NAFLD is characterized by the excessive accumulation of triglycerides (TGs) in hepatocytes and encompasses a spectrum of chronic liver abnormalities, ranging from simple steatosis (NAFL) to steatohepatitis (NASH), significant liver fibrosis, cirrhosis, and hepatocellular carcinoma. Although the molecular mechanisms that cause the progression of steatosis to severe liver damage are not fully understood, metabolic-dysfunction-associated fatty liver disease is strong evidence that mitochondrial dysfunction plays a significant role in the development and progression of NAFLD. Mitochondria are highly dynamic organelles that undergo functional and structural adaptations to meet the metabolic requirements of the cell. Alterations in nutrient availability or cellular energy needs can modify mitochondria formation through biogenesis or the opposite processes of fission and fusion and fragmentation. In NAFL, simple steatosis can be seen as an adaptive response to storing lipotoxic free fatty acids (FFAs) as inert TGs due to chronic perturbation in lipid metabolism and lipotoxic insults. However, when liver hepatocytes’ adaptive mechanisms are overburdened, lipotoxicity occurs, contributing to reactive oxygen species (ROS) formation, mitochondrial dysfunction, and endoplasmic reticulum (ER) stress. Impaired mitochondrial fatty acid oxidation, reduction in mitochondrial quality, and disrupted mitochondrial function are associated with a decrease in the energy levels and impaired redox balance and negatively affect mitochondria hepatocyte tolerance towards damaging hits. However, the sequence of events underlying mitochondrial failure from steatosis to hepatocarcinoma is still yet to be fully clarified. This review provides an overview of our understanding of mitochondrial adaptation in initial NAFLD stages and highlights how hepatic mitochondrial dysfunction and heterogeneity contribute to disease pathophysiology progression, from steatosis to hepatocellular carcinoma. Improving our understanding of different aspects of hepatocytes’ mitochondrial physiology in the context of disease development and progression is crucial to improving diagnosis, management, and therapy of NAFLD/NASH.

## 1. Introduction

Non-alcoholic fatty liver disease (NAFLD) is a spectrum of fatty liver phenotypes that do not result from alcohol consumption abuse (i.e., ≤30 g alcohol/day in men; ≤20 g alcohol/day in women), viral or autoimmune factors, or drug exposure. Global prevalence of NAFLD is around 25–29% and is closely associated with multiple metabolic disorders such as type 2 diabetes mellitus (T2M), obesity, hypertension, and hyperlipidemia, but a contribution of the hereditary component has also been demonstrated [1]. The diagnosis of this disease requires evidence of an excessive accumulation of TG in the hepatocytes while excluding other factors that also can lead to development of hepatic steatosis. NAFLD can range from NAFL or simple steatosis, a more benign stage, to NASH, which can progress to cirrhosis and hepatocellular carcinoma (HCC) [2,3]. The pathophysiology of NAFLD/NASH is multifactorial, and the progression to advanced forms remains unclear. Notwithstanding, two-hit or, more recently, the multiple-hit hypothesis is the most widely accepted explanation for NAFLD development. Accordingly, hepatic steatosis (first-hit) can progress to further stages due to several second hits (mitochondrial dysfunction, oxidative stress, proinflammatory cytokines, or gut-derived bacterial endotoxins).

Mitochondria play an important role in maintaining homeostasis in the liver and, consequently, are closely involved in the development of NAFLD disease. Although mitochondria counteract lipotoxic insults in the initial stage of the disease, prolonged uncontrolled stimulation of basal reactive oxygen species (ROS) production or failure of antioxidant defenses to neutralize them results in oxidative stress and hepatocyte injury [4]. Still, some aspects of hepatocytes’ mitochondrial physiology in NAFLD remain unclear.

This review postulates that multiple pathways involved in regulating hepatocytes’ mitochondrial physiology to maintain hepatic mitochondrial mass, integrity, and function are altered differently during NAFLD progression. This review summarizes the current evidence that links mitochondrial (dys)function with NAFLD pathophysiology, discriminating them in different disease stages. This knowledge of the mitochondrial physiology of hepatocytes and associated signaling pathways involved in disease development and progression will decisively impact diagnosis, management, and therapy of NAFLD/NASH.

## 2. NAFLD Disease

### 2.1. Epidemiology

The incidence and prevalence of NAFLD is increasing worldwide. Statistically, the global prevalence of NAFLD is around 25–29%, with the lowest rate in Africa (13%) and the highest in Southeast Asia (42%) [5]. In Europe, the prevalence of NAFLD is approximately 24%, with notoriously higher rates in Southern than Northern Europe [6,7]. Additionally, a time-dependent uptrend in NAFLD prevalence is documented (from 25% between 1999 and 2005, to 28% between 2006 and 2011, and 34% (32–36) between 2012 and 2017) [5]. NAFLD incidence is higher in men than in women (37% vs. 23% and more common in older populations (age ≥ 45 years) than in younger populations (age < 45 years; 32% vs. 27%) [5]. Although NAFLD is strongly associated with obesity episodes, lean NAFLD is also a health concern. From the NAFL patient population, 59% progress to NASH, of whom 41% develop fibrosis. Additionally, 40% of the patients with fibrosis become cirrhotic [8]. NASH has a global prevalence estimated between 3% to 5%. Still, NASH is responsible for 18% of all HCC cases in the USA, which corresponds to an 8-fold increase from 2002 to 2017 [9]. Concerningly, NAFLD is a rising cause of HCC worldwide.

### 2.2. Risk Factors

NAFLD is mainly the result of unhealthy dietary habits such as high caloric intake and fructose consumption combined with sedentary lifestyle [9]. A meta-analysis showed that 51% of patients with NAFLD were obese, 23% had type 2 diabetes mellitus (T2DM), 69% had hyperlipidemia, 39% had hypertension, and 42% had metabolic syndrome [5]. Obesity, defined by the World Health Organization (WHO) as a body mass index (BMI) higher or equal to 30, is proportional to the rising prevalence of NAFLD [10]. T2DM is another critical risk factor for NAFLD and NASH. The manifestation of NAFLD among people with diabetes is 56%, whereas the overall prevalence of NASH in people who have diabetes type II (T2DM) is around 37% [7]. Likewise, dyslipidemia is a risk factor that is characterized by exacerbated triglycerides (TGs) and low-density lipoprotein cholesterol (LDL-C) levels and by diminished high-density lipoprotein cholesterol (HDL-C) concentrations. Hypertension is also recognized as a major (cardio)metabolic risk for NAFLD, since 50% of hypertensive patients have the disease [11,12,13].

In addition, some genetic contributions to NAFLD have been documented, accounting for between 20–70% of NAFLD development (Table 1). Although five major variants in genes associated exclusively with NAFLD, such as patatin-like phospholipase domain-containing 3 (*PNPLA3*), transmembrane 6 superfamily member 2 (*TM6SF2*), glucokinase regulator (*GCKR*), membrane-bound O-acyltransferase domain-containing 7 (*MBOAT7*), and hydroxysteroid 17-beta dehydrogenase 13 (*HSD17B13*), have been described, rare variants were also reported in NAFLD patients [14]. *PNPLA3* gene, responsible for encoding adiponutrin (ADPN) protein and located on the lipid droplets of hepatocytes, acts as a hydrolase towards to TGs and transacylase at polyunsaturated fatty acids (PUFAs) in phospholipids. The replacement of glutamic acid with lysine at position 434 (PNPLA3 148M, rs2294918) impairs ubiquitylation and proteasomal degradation, resulting in lipid droplet accumulation and NAFLD development [15]. TM6SF2, highly abundant in the liver and small intestine, is mainly localized in the endoplasmic reticulum (ER) and the ER–Golgi intermediate compartment (ERGIC) and plays a regulatory function in liver fat metabolism, orchestrating triglyceride secretion and hepatic lipid droplet content. A nonsynonymous mutation in TM6SF2 (TM6SF2 E167K, rs58542926) leads to a substitution of a glutamine by a lysine at residue 167 that promotes protein degradation [16]. TM6SF2 E167K variant can contribute to liver steatosis due to abnormal TG synthesis or low secretion of VLDL-TG or a combination of both as it impairs the second stage of lipidation of VLDLs, affecting the incorporation of PUFAs into serum TGs and liver phosphocholines (PCs) [17]. The *GCKR gene* encodes glucokinase regulatory protein (GCKRP), which plays a huge role in preserving plasma glucose homeostasis and metabolic traits. A common missense variant (rs1260326) resulting from a proline-to-leucine substitution at position 446 (P446L) showed impaired regulation of fructose-6-phosphate levels that turned out to increase GCK activity [18] continually. In fact, augmented liver GCK activity is known to stimulate glycolytic flux, which promotes hepatic glucose metabolism and increased concentrations of malonyl-CoA, leading to hepatic fat storage due to β-oxidation blocking ability of malonyl-Coa [19]. *HSD17B13* gene expression is confined to the liver, in particular hepatocytes. It is a lipid droplet (LD)-associated protein involved in the insulin-regulated lipogenic transcription factor steroid-responsive element-binding protein 1c (SREBP1c) and upregulated in human NAFLD livers [20,21]. Additionally, it catalyzes multiple substrates such as steroids, lipids including leukotriene B4 and 12(R)-hydroxyeicosatetraenoic acid, and retinol. A protein-truncating variant of HSD17B13 (rs72613567) resulted in lower risks of developing chronic hepatic inflammation and ballooning as well as fibrosis [22]. Notably, this variant did not prevent steatosis, and its protective effect may be determined by the coexistence of other genetic variants such as PNPLA3 rs2294918 and TM6SF2 rs58542926 [21]. The *MBOAT7 gene* is highly expressed in circulating monocytes and lymphocytes codifying the MBOAT7 enzyme that participate in the phospholipid acyl-chain remodeling of the membranes. MBOAT7 catalyzes a desaturation of the second acyl-chain of phospholipids by transferring a PUFA in the form of acyl-CoA to lysophosphatidylinositol (LPI) and other lysophospholipids. Since it uses arachidonoyl-CoA as a substrate, MBOAT7 can regulate the levels of free arachidonic acid and consequently eicosanoids that are potent triggers for hepatic inflammation and fibrosis. The MBOAT7 variant (rs641738 C>T MBOAT7/TMC4) reduces the levels of the MBOAT7 protein in the liver and predisposes to advance forms of NAFLD, mainly by modifying the hepatic levels of phosphatidylinositol (PI) and LPI and stimulating hyperinsulinemia and hepatic IR [23,24]. Many other rare genetic variants related to NAFLD have been described. Among them, protein phosphatase 1 regulatory subunit 3B (*PPP1R3B*, rs4240624), autophagy-related 7 (*ATG7*, rs143545741), immunity-related GTPase M (*IRGM*, rs10065172), lipin 1 (*Lpin1*, rs13412852), uncoupling protein 2 (*UCP2,* rs695366), mitochondrial amidoxime reducing component 1 (*MARC1*, rs2642438), interferon-l4 (*IFNL4*, rs368234815), MER proto-oncogene, tyrosine kinase (*MERTK*, rs4374383), superoxide dismutase 2 (*SOD2*, rs4880), and Kruppel-like factor 6 (*KFL6*, rs3750861) were reported in NAFLD patients [25].

In summary, rare genetic variants can contribute to NAFLD development, but the lack of information on the role of rare variants, as well as structural variation, gene-by-gene-interaction, and gene-by-environment interaction emphasize the need to improve our understanding as they likely contribute to the disease development and may impact NAFLD clinical diagnosis.

### 2.3. Pathophysiology

NAFLD pathophysiology is characterized by excessive fat in the liver, specifically when at least 5% of hepatocytes exhibit lipid droplets (LDs) that surpass 5–10% of total liver weight in patients. The histological hallmark of NAFLD is steatosis, the accumulation of hepatic TGs, which relies on the acinar architecture and is classified according to the percentage of liver parenchyma containing steatotic hepatocytes: 0–33%, 33–66%, or >66% [34]. The increasing FFA flux into the liver from lipolysis (the hydrolysis of FFAs and glycerol from triglyceride) within adipose tissue, dietary sources, and de novo lipogenesis (DNL), combined with an imbalance in its oxidation or secretion, leads to hepatic steatosis [35]. Once in the liver, FFAs are metabolized either through β-oxidation or re-esterification to TGs and storage as LD or wrapped and exported as very low-density lipoproteins (VLDLs). In NAFLD patients, 60% of liver TG content is derived from nonesterified fatty acid pool (NEFA), 26% from DNL, and 15% from the diet, while in healthy individuals DNL only contributes to <5% of hepatic TG formation [36].

Classically, progressive liver steatosis, if not treated, may advance to NASH and fibrosis. The NASH histological diagnostic criteria include steatosis, hepatocellular injury, and lobular inflammation, with or without fibrosis. As the subsequent impairment of insulin signaling in the adipose tissue results in the suppression of lipolysis and inflammation, and the adaptive mechanisms in the liver are overburdened, lipotoxicity occurs, a process that can contribute to ROS formation, mitochondrial dysfunction, and ER stress. Elevated proinflammatory adipocytokines such as TNF-α, IL-6, and IL-1β produced by adipose tissue activate the immune system in the liver and act as precursors to the development of insulin resistance and compensatory hyperinsulinemia [37]. The cellular damage caused by the combination of these insults leads to the transition from NASH to fibrosis [38]. Once fibrosis progresses, hepatic architectural remodeling occurs, and hepatocellular injury occurs in the form of ballooning, the formation of apoptotic bodies, and lytic necrosis. Hepatocyte ballooning describes the presence of enlarged, swollen hepatocytes, with rarefied cytoplasm that may have a reticulated appearance or contain Mallory–Denk bodies. The need to replace dead cells leads to the activation of liver regeneration and fibrogenesis, extracellular matrix (ECM) production, and augmenting collagen, promoting liver fibrosis [39]. Approximately 25% of NAFL-diagnosed patients develop advanced fibrosis during a relatively short follow-up period (median: 6.7 years; mean: 3.3 years), which reflects an active disease predisposing denominator to both liver- and non-liver related morbidity and mortality. Advanced fibrosis increases the likelihood of cirrhosis and, ultimately, may develop into hepatocellular cancer and liver failure due to impaired liver regeneration brought on by unsuccessful attempts to restore healthy liver architecture [39,40,41].

## 3. Two-Hit or Multiple-Hit Hypothesis

The full understanding of the mechanisms underlying the development of NAFLD is of extreme importance, although the pathophysiology is complex and incompletely understood. The ‘two-hit’ hypothesis is now obsolete, as it is inadequate to explain the several molecular and metabolic changes that take place in NAFLD. The “multiple-hit” hypothesis considers multiple insults acting together on genetically predisposed subjects to induce NAFLD and provides a more accurate explanation of NAFLD pathogenesis. According to this hypothesis, the first hit consists of hepatic fat accumulation that occurs in response to increased fat synthesis and delivery, decreased fat export, and/or diminished fat oxidation [42].

Hepatic steatosis can progress to further stages due to “second hits” such as mitochondrial dysfunction, proinflammatory cytokines (IL-1β, TNF-α, and IL-6) and adipokines (adiponectin and IL-37), ER stress, and gut-derived bacterial endotoxins [43,44,45,46,47]. The theory evolved as new evidence showed that NAFLD may be a consequence of parallel “multi-hits” [48]. In this context, insulin resistance leads to enhanced lipogenesis and increased uptake of FFAs into the liver, which predispose the liver to injury by “multiple parallel hits” (oxidative damage, activation of fibrogenic pathways, activation of hepatic stellate cells (HSCs), altered expression of adipokines) leading to NASH and fibrosis. Mitochondrial dysfunction has been described as a crucial driving force in NAFLD progression. In the sections below, the role of mitochondria in NAFLD progression will be discussed.

## 4. Mitochondrial (Dys)Function in NAFLD

### 4.1. Hepatic Mitochondrial Populations: Implications for NAFLD

**Figure 1:**
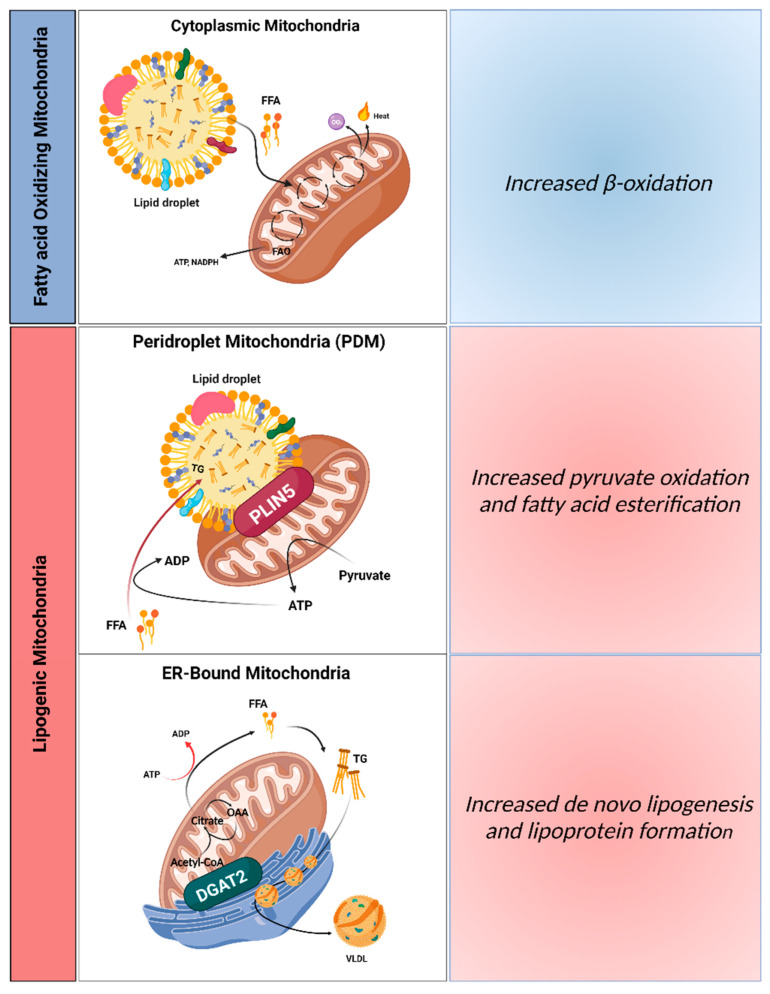
**Distinct mitochondrial populations in hepatocytes.** Mitochondria attached to different organelles can have distinct roles, reinforcing the hypothesis that not all mitochondria in the same cell are homogeneous. This concept supports the assumption that some mitochondria in hepatocytes can be specialized in synthesizing lipids, while other mitochondria can oxidize lipids. The functional segregation of mitochondria can be determined by their anchorage to specific organelles, which prevents motility and thus fusion between the different subpopulations. In this context, it is believed that three mitochondrial populations exist in hepatocytes: (1) cytosolic mitochondria, which are responsible for fatty acid oxidation, production of ketone bodies, and ureagenesis to support glucose production; (2) mitochondria attached to lipid droplets, namely peridroplet mitochondria (PDM), which promote the esterification of fatty acids into triglycerides; and (3) ER-anchored mitochondria, responsible for fatty acid synthesis, lipoprotein assembly, and excretion. VLDL, very low-density lipoprotein; PLIN5, perilipin 5; DGAT2, diacylglycerol O-acyltransferase 2; ER, endoplasmic reticulum; OAA, oxaloacetate. Created with BioRender.com (accessed on 5 February 2023).

The number and type of mitochondrial structures diverge from tissue to tissue, depending on their metabolic state. However mitochondrial heterogeneity can occur even within the same tissue, including liver. Experimental data using intact livers and isolated liver mitochondria revealed an increment in mitochondrial oxidative function induced by simple steatosis [49], even though in primary hepatocytes and human hepatoma cell lines subjected to lipotoxicity insults opposing effects were observed [50,51]. In a study in brown adipose tissue (BAT), two segregated populations of mitochondria, the peridroplet mitochondria (PDM) and the cytosolic mitochondria (CM), were detected [52]. Mitochondria bound to lipid droplets (LDs), namely PDM, can stimulate TG synthesis and expand LDs in a process involving protein perilipin 5 (PLIN5) [52]. Similarly, hepatic PLIN5 retains the dietary excess of FAs in LDs and blocks lipolysis, which protect hepatic from IR damage induced by HFD feeding [53]. Likewise, it was proposed that hepatocytes possess segregated mitochondria, which would be dedicated to sustaining dietary FA esterification into TGs but also to producing new FAs and assembling lipoproteins in the ER (Figure 1). As TG, cholesterol, and phospholipids can be carried inside of this type of lipoprotein, which allows their efflux from hepatocytes to plasma, a new segregated mitochondrial population, ER-anchored mitochondria, were suggested [54]. In this scenario, ER-anchored mitochondria showed inhibited CPT-1 activity and are probably responsible for lipoprotein assembly and de novo FA synthesis. Peridroplet mitochondria (PDM) have a limited capacity to execute FAO. On other hand, cytosolic mitochondria (CM) are proposed to be more involved in oxidizing FAs. Since hyperinsulinemia stimulates lipogenesis, it is possible that the increase in ER–mitochondria contacts with IR and that simple steatosis is a consequence of hyperinsulinemia [54]. Notably, overexpression of PLIN5 and consequently PDM do not augment de novo lipogenesis or the assembly of lipoproteins in the ER, as observed by the unchanged VLDL export and plasma lipid levels [55]. Furthermore, diacylglycerol o-acyltransferase 2 (DGAT2) and not PLIN5 appears to be the mediator in mitochondria segregation and ER-anchored lipogenic mitochondria generation [56], as demonstrated by the reduction on steatosis and hypertriglyceridemia upon DGAT2 ablation [57]. Functionally, DGAT2 catalyzes the last step of TG synthesis and is found in mitochondria-associated membranes (MAMs).

These segregated subpopulations of mitochondria can be independent and have diverse oxidative functions, which may explain why the tricarboxylic acid (TCA) cycle fluxes and mitochondrial fatty acid β-oxidation (FAO) rates increase in NAFLD (in animal models). This process is not always observed in isolated liver mitochondria by the inability to access in ex vivo experiments full mitochondrial oxidative function. Although studies on distinct mitochondrial sub-populations roles in NAFLD are still scarce, and their role in metabolic dysregulation remains poorly understood, the idea that liver ER-anchored mitochondria and PDM are functionally disconnected from cytosolic mitochondria can explain the mitochondria oxidative function heterogeneity in NAFLD.

### 4.2. Adaptation (Hormesis) vs. Maladaptation (Dysfunction)

Multiple pathways can be involved in the regulation of hepatocytes’ mitochondrial physiology in order to maintain hepatic mitochondrial mass, integrity, and function. Alterations in mitochondria-associated signaling pathways decisively impact development and progression of NAFLD/NASH. Hormesis, a “biphasic dose response” [58], is defined as a beneficial or stimulatory effect caused by exposure to low doses of an agent capable of stimulating adaptive responses in cells and organisms to preserve homeostasis, promoting health and longevity. More recently, the “mitohormesis” concept was introduced as the hormetic response that promotes health and vitality upon sublethal mitochondrial stress [59]. Activation of ROS-mediated (mainly H_2_O_2_) cell signaling pathways is crucial for mitohormesis regulation [60,61].

**Figure 2:**
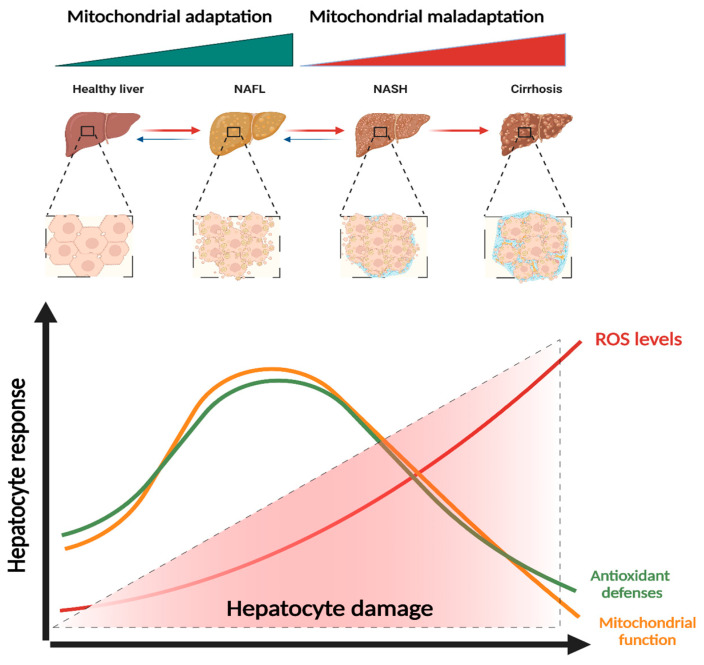
**Mitochondrial adaptation and maladaptation in NAFLD progression.** Different stages of Non-alcoholic fatty liver disease (NAFLD), comprising non-alcoholic fatty liver (NAFL), non-alcoholic steatohepatitis (NASH), cirrhosis, and hepatocellular carcinoma (HCC). During NAFLD progression, mitochondria increase their function in order to counteract lipotoxic insults. However, uncontrolled stimulation of basal ROS production or failure of antioxidant defenses to neutralize ROS results in exacerbated ROS levels that lead to oxidative stress and culminate in hepatocyte injury. ROS, reactive oxygen species. Created with BioRender.com (accessed on 5 February 2023).

NAFLD patients showed increased serum levels of antioxidant enzymes such as SOD, GPx, and GSH in an initial stage of NAFLD [62], indicating a possible adaptation mechanism to mild increase in oxidative stress (Figure 2). Activation of the SIRT1-PGC-1α pathway, either by an alteration in AMP/ATP ratio or AMPK activation and fluctuation in NAD^+^ levels, can upregulate mitochondrial energy metabolism and biogenesis [63]. In mouse models, it was observed that SIRT1 overexpression attenuated HFD-induced liver steatosis and inflammation by inhibiting CD36 expression and the NF-κB signaling pathway [64].

Proteotoxic signals, such as mitochondrial unfolded protein response (UPR^mt^), are also involved in hormetic processes. UPR^mt^ alteration was observed in mouse studies and obese humans [65,66,67], constituting a stimulus that may trigger UPRer, UPRmt, or both. Moreover, the downregulation of the TOR signaling pathway led to an early increase in mtROS levels [68]. In liver tumors, mTOR activation has been associated with NASH in both mouse models and humans [69]. The nuclear factor erythroid-derived 2-like 2 (Nrf2, NFE2L2) also contributes to hormetic induction of increased stress resistance [70]. Specific overexpression of hepatic Nrf2 in HFD-fed mice protects against OxS induced by prolonged methionine- and choline-deficient (MCD) exposure [71], while Nrf2 deletion results in progression from simple steatosis to NASH [72]. Interestingly, liver macrophages showed diminished levels of Nrf2 [73].

Mitochondrial homeostasis is assured by the successful removal of physiological ROS through endogenous antioxidant mechanisms as well as by assisting metabolic adaptations that prevent substrate supply to the TCA cycle. However, amplified and chronic mitochondrial ROS production and redox processes that damage mitochondria can be part of a deleterious cycle as mitochondria can continuously produce more ROS (Figure 2) [74]. While mitochondria are the preferential site of ROS production, particularly under pathological conditions, other mitochondrial enzymes such as glycerol 3-phosphate dehydrogenase and 2-oxoglutarate dehydrogenase can also contribute to a decay of mitochondrial homeostasis [75] and induce a stress signaling response and mitochondrial dysfunction. Liver tissues from patients with NASH exhibited high levels of mtROS and mtDNA damage [76]. mtDNA depletion and augmented levels of 8-hydroxy-20-deoxyguanosine (8-OHdG), an oxidized form deoxyguanosine, were reported in NAFLD [77]. Critical regulators of mitochondrial metabolism and biogenesis such as TFAM, Nrf2, and PGC-1α showed reduced expression levels in NAFLD [75]. Due to the high FA hepatic influx, patients with NAFLD have elevated mitochondrial FAO and TCA cycle turnover, which result in a consistent high source of reducing equivalents to the ETC. Although increased mitochondrial FAO characterizes NAFLD, in NAFLD patients and high-fat-fed mice, the upregulation proliferator-activated receptor-α (PPAR-α) and ACOX genes and higher levels of peroxisomal-related proteins in livers were detected [78,79]. The increased content of oxidized cardiolipin (CL), a phospholipid present in the inner mitochondrial membrane, contributes to mitochondria dysfunction by decreasing ETC complex activity and promoting the mPTP opening [80]. This process seems to involve ALCAT1, a lyso-CL acyltransferase.

Ceramides are crucial cell membranes components that can act as signaling molecules coordinating various cellular processes such as proliferation and apoptosis. In response to HFD feeding of mice, ceramide synthesis can be exacerbated, leading to the decrease in mitochondrial respiration and contributing to NASH. Sphingolipid ceramide 16:0 directly decreases mitochondrial FAO in hepatocytes from steatotic mice, accompanied by reduced hepatic insulin signaling and hyperglycemia [81]. Notably, the data from cohorts of NASH patients presented a strong correlation between serum and liver ceramide levels [82]. Exacerbated hepatic ceramide content is linked to diminished mitochondrial oxidative capacity [83]. Ceramides accelerate the synthesis of ganglioside GD3 at the ER, promoting its translocation to mitochondria, where it enhances OxS by stimulating superoxide anion production at complex III [84]. Steatotic livers from ob/ob mice also showed exacerbated levels of TNF-α and FFAs [4,85], together with decreased ETC coupling, as demonstrated by the higher proton leak and subsequent decrease in ATP synthesis [86].

The activation of pathways involving mitogen-activated protein kinases (MAPKs) play a critical role in the development of liver diseases and injuries, such as steatosis, NASH, fibrosis and hepatocarcinoma [87]. Oxidative stress prompts a progressive and irreversible escalation of oxidative damage that markedly influences critical aspects of mitochondrial physiology (oxygen consumption, ATP and ROS production, FAO, autophagy, among others), which might contribute to NAFLD disease progression. However, the mitochondrial capacity breaking point, i.e., the sequence by which signaling pathways transform functional mitochondria into dysfunctional mitochondria, is unknown.

## 5. Mitochondrial (Dys)Function in NAFLD Progression

### 5.1. Mitochondrial Function in Simples Steatosis

**Figure 3:**
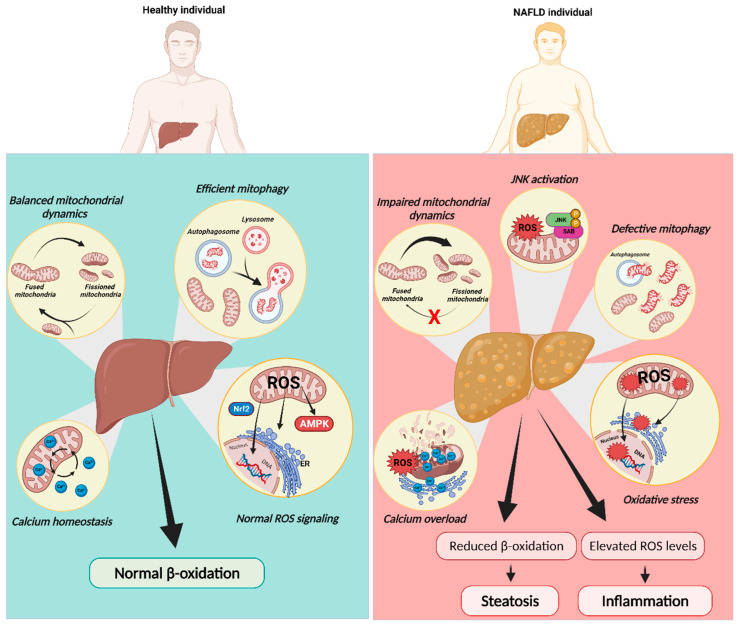
**Mitochondrial dysfunction in NAFLD progression.** Mitochondria rely on diverse mechanisms to preserve their function including dynamics, redox signaling, mitophagy, and calcium homeostasis. In contrast to a healthy liver, mitochondria in NAFLD were reported to be fragmented and overloaded with calcium, with decreased oxidative capacity and increased ROS production, which cause JNK activation. JNK activation itself can induce these same defects in mitochondrial function, constituting a feed-forward cycle of mitochondrial dysfunction. Mitochondrial dysfunction in NAFLD was also explained by defective mitophagy. The decrease in fatty acid oxidation (FAO) caused by this compromise in mitochondrial function may induce FA accumulation in hepatocytes while impairing insulin signaling. ER, endoplasmic reticulum; JNK, c-Jun NH2-terminal kinase; SAB, SH3 homology associated BTK-binding protein; ROS, reactive oxygen species. Created with BioRender.com (accessed on 5 February 2023).

In simple steatosis, the dysregulation of lipid and glucose metabolism occurs mainly in the presence of a high intake of high-caloric fat diets, which results in accumulation of TGs and FFAs in the liver. The overall process contributes to modifying the mitochondrial proteome [88]. In response to fat overload, hepatocytes increase fatty acid oxidation (FAO) processes, followed by increasing the TCA cycle and OXPHOS to avoid hepatic lipid burden. AMPK triggers catabolic pathways (e.g., fatty acid and glucose oxidation pathways) by inducing the activation of PGC-1α (Figure 3) [89]. When PGC-1α is activated, it powerfully coordinates gene expression that stimulates mitochondrial fatty oxidation. Through interaction with PPAR-α, PGC-1α induces the expression of several enzymes involved in fatty acid-metabolism, including carnitine palmitoyltransferase-1 (CPT-1) and acyl-CoA dehydrogenases [76]. The carnitine palmitoyltransferase system is an essential step in the β-oxidation of long chain fatty acids, as CPT-1 catalyzes the import of FFAs into the mitochondria. Clinically, CPT-1 activation can increase NAFLD biomarkers in patients, as demonstrated by the decrease in serum levels of AST, ALT, bilirubin, and mtDNA [90]. Moreover, the observed alterations in the mitochondrial composition may reflect the adaptation to the chronic rise in gluconeogenesis and intrahepatic lipid management induced by NAFLD, leading to accumulation of mitochondrial ATP and TCA cycle intermediates. Studies in the fatty liver of human and mice demonstrated that mitochondrial pyruvate oxidation and TCA cycle flux are elevated in the fasted stage and that ketogenesis does not follow the same tendency [91]. The mismatch of mitochondria between high TCA fluxes and decreased OXPHOS activity alongside high rates of FAO could be the key pathogenic mechanism in NAFLD. In fact, impaired ketosis can raise TCA flux in NAFLD, which increases acetyl-CoA available to the TCA cycle. Notably, in HFD mice, the deletion of the mitochondrial pyruvate carrier 1 (MPC1), which decreases TCA fluxes, led to diminished hepatic glucose production and inflammation [92,93].

Several in vitro studies mimicking NAFLD conditions as well as mice subjected to HFD showed impaired mitophagy, culminating in increased fat accumulation, elevated OxS, and inflammation [94,95] (Figure 3). HFD-fed mice presented exacerbated expression levels of ALCAT1 and defective mitophagy in isolated hepatocytes, which were restored after genetic ablation of ALCAT1, strengthening the importance of maintaining mitochondrial morphology and mtDNA integrity [96]. Knockout mouse studies showed that BNIP3, an important regulator of hepatic lipid metabolism, led to augmented lipid synthesis, mainly by reducing AMPK activity and increasing expression of lipogenic genes. In addition, decreased β-oxidation was reported [97]. Finally, it was found that peroxiredoxin 6 (PRDX6), an antioxidant PRDX family member, can translocate to dysfunctional mitochondria upon increased ROS production, where it plays a crucial role in the initial stage of mitophagy by controlling ROS homeostasis. Moreover, PRDX6 antagonizes the positive feedback loop between lipid accumulation and ROS production through the regulation of mitochondrial antioxidant function and β-oxidation to maintain mitochondrial integrity [98]. Although OxS is clearly documented as a trigger of NAFLD progression, the role of mitochondria ROS in severity transition remains controversial. In fact, several authors showed no alterations in ROS levels of isolated liver mitochondria from mice with the NAFL phenotype [99,100] Peroxisomes are important organelles in fat metabolism, also being responsible for ROS generation. The knowledge of how these two organelles communicate is very limited. Further studies on contact sites between mitochondria and peroxisomes and the sub-localization of ROS in steatosis are still needed.

### 5.2. Mitochondrial Function in NASH and Non-Alcoholic Fibrosis (NAF)

In liver biopsies of NASH patients, mitochondria presented ultrastructural abnormalities [101]. In NAFLD, the sequential events of increased FFAs and de novo lipogenesis (DNL) and accumulation of TGs induce adaptations of mitochondrial oxidative metabolism. However, even a dynamic organelle such as mitochondria cannot infinitely protect cells against lipotoxicity with the excessive deposition of FFAs.

**Figure 4:**
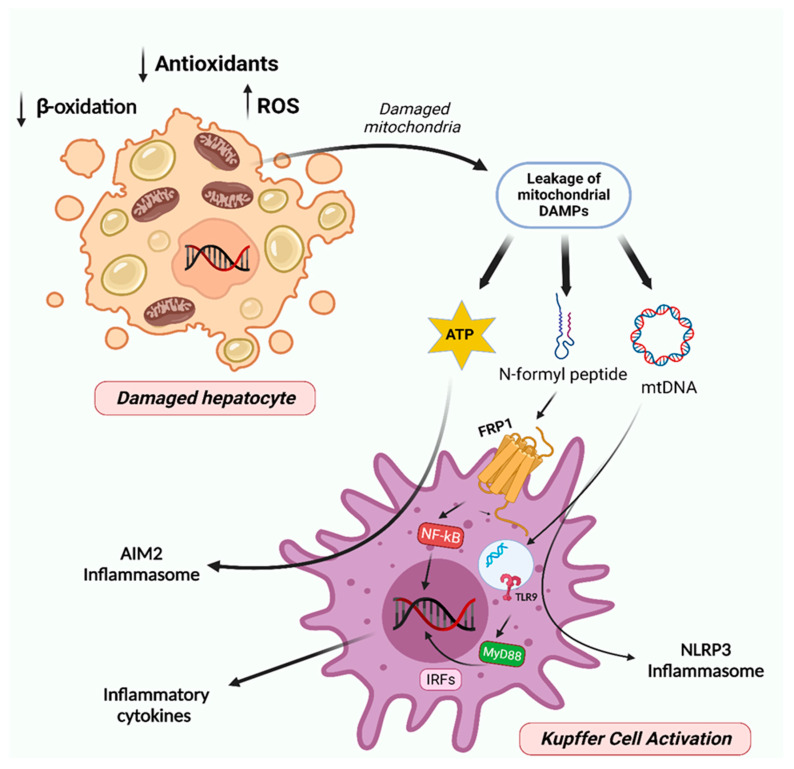
**Mitochondria involvement in NAFL progression to steatohepatitis and fibrosis**. The augmented accumulation of damaged/dysfunctional mitochondria within hepatocytes results in cell necrosis and induces the leakage of mitochondrial DAMPs, such as mtDNA, N-formyl peptides, and ATP. Further, these signals trigger the activation of toll-like receptor 9 (TLR9) and formyl peptide receptor 1 (FPR1), which in turn activates the IRFs and NF-kB and thereby the production of inflammatory cytokines. mtDNA and ATP also activate the inflammasomes NLRP3 and AIM2, respectively. Multiple inflammatory cytokines and the activation of inflammasomes provide a chronic inflammatory milieu, which contributes to the development of steatohepatitis and fibrosis. Created with BioRender.com (accessed on 5 February 2023).

NAFLD progression is amplified by inhibition of CPT-1, diminished mitochondrial FAO, and continuing ATP depletion, which is promoted by increased hepatic expression of uncoupling protein-2 (UCP2) [102]. Subsequently, increased ROS levels also contribute to TNF synthesis and several other cytokines, which can cause apoptosis and necroptosis. DNA-enriched mitochondria-derived danger-associated molecular patterns (DAMPs) (Figure 4) produced by damaged hepatocyte mitochondria, activate NOD-like receptor family pyrin domain-containing 3 (NLRP3) and other innate immune system inflammasomes through pattern recognition receptors such as toll-like receptors (TLRs) (Figure 4) [103,104,105]. Moreover, OxS and lipid peroxidation participate in inflammatory response by activating NF-κB and the production of proinflammatory cytokines (TNF-α, IL-1β, Il-6, and IL-8), which culminate in apoptosis and necrosis in hepatocytes [106,107]. The combination of events comprising ROS-associated lipid peroxidation, mitochondrial DAMPs, and activation of caspases promote chronic liver injury via intrusion of inflammatory cells [106,108]. Studies in HFD-fed mice showed that mtDNA released by injured hepatocytes activate TLR9 on Kupffer cells (KCs) and HSCs, stimulating the innate immune as well as fibrogenic responses [108]. Transition from steatosis to NASH is accelerated by mitochondrial cholesterol deposition, as it causes alterations in mitochondrial membrane permeability and impairs protein transport from mitochondria to cytosol and vice versa [109]. In fact, mPTP opening episodes appear critical in hepatocyte cell death [110]. This process seems to be correlated with overexpression of StARD1, a mitochondrial cholesterol-transporting polypeptide involved in the trafficking of cholesterol to IMM [109]. Although the impact of autophagic dysregulation in NAFL progression to NASH is still under debate, several mechanisms of autophagy impairment have been described in NAFLD. Decreased in ATG7 expression levels and increased activation of proteases, such as calpain-2, which can diminish autophagy proteins levels, namely ATG3, ATG5, Beclin1, and ATG7, were found to reduce autophagy flux in NAFLD [111]. Moreover, accumulation of LC3-II and p62 were detected in NASH patients, and their increase positively correlates with disease severity [112]. Silencing of macrophage stimulating 1 (MST1), a cell survival regulator, stimulated PINK1/Parkin-mediated mitophagy and counteracted HFD-induced liver injury [113]. Moreover, impairment of MFN2 activity induced by JNK activation during inflammation could trigger mitophagy defects, as MFN2 supports the formation of autophagosomes [114]. OPA1 ablation in the liver led to the recovery of mitochondrial homeostasis and reduced the accumulation of mitophagy intermediates, which could contribute to alleviation of MCD diet-induced liver damage in mice [115]. Patients diagnosed with NASH showed a negative correlation between cholesterol content and mitochondrial GSH levels [116], probably resulting from an impairment in the mitochondrial GSH transport system. Finally, the high levels of cholesterol in obese mice also seem to stimulate TNF-α and Fas-induced apoptosis in hepatocytes, contributing to mitochondrial GSH ablation [117]. Nutritional and genetic NASH mouse models exhibited an increased expression in the levels of SOD2, paralleled by a significant impairment in its activity, which may reflect sensitivity to OxS in the later NAFLD stages [118]. Interestingly, hepatocyte-specific deletion of GPx1 resulted in diminished hepatic lymphocytic infiltration, inflammation, and liver fibrosis in mice with the NASH phenotype [119]. As result of GPx1 ablation, H_2_O_2_ signaling led to protein-tyrosine phosphatase 1B (PTP1B) inactivation. Hyperactivation of PTP1B promotes IR and steatosis by dephosphorylating the insulin receptor and increasing SREBP-1c activity [120]. Moreover, heme oxygenase 1 (HMOX1) deletion in hepatocytes increased H_2_O_2_-mediated PTP1B inactivation, protecting mice from NAFLD and hyperglycemia [121].

Summing up, the transition from NAFL to NASH is accompanied by a decrease in mitochondrial plasticity, resulting in decline in ketogenesis, TCA turnover, OXPHOS capacity, and ATP production. As mitochondria are important immune-cell mediators, at this stage, the release of mitochondrial danger signals by damaged mitochondria impacts inflammation and leads to the recruitment and activation of KCs. Several mitochondrial damage-associated molecular patterns (DAMPs) containing intact mitochondria and high levels of mitochondrial oxidized DNA enclosed in microparticles of hepatocyte origin were already identified in plasma from patients with NASH [122]. However, it remains unclear whether DAMPs play a role in the development of extra-hepatic complications associated with NAFL/NASH.

### 5.3. Mitochondria Function in Hepatocellular Carcinoma (HCC)

The last stage of liver disease progression can be hepatocellular carcinoma (HCC). An increase in the prevalence of HCC was observed in NAFLD patients, in which tumor development occurred in non-cirrhotic livers, indicating that HCC can still develop in the NASH stage [123]. Lipotoxicity, inflammation, OxS, mitochondrial dysfunction, and ER stress are factors that produce an ideal environment for tumor promotion. Lipid-induced alterations in intracellular Ca^2+^ homeostasis play a crucial role in the progression of the disease. In fact, ob/ob mice fed with a high-fat diet exacerbated lipid accumulation causing ER stress and impaired ER–mitochondria connections in steatotic hepatocytes [124]. Moreover, changes in Ca^2+^ channels and transporters promote decreased Ca2+ concentration in the ER and increased Ca^2+^ concentration in the cytoplasm and in the mitochondrial matrix. As a consequence of the increase in ROS levels and subsequent mitochondrial damage, oxidative damage and mutations of both nDNA and mtDNA induce aberrant activation of proliferative pathways and inhibition of oncosuppressors [124]. Moreover, increased ROS levels induced by dysregulation of Ca^2+^ homeostasis can activate Nrf2 signaling pathways. Although Nrf2 is a transcription factor essential to protecting the liver from OxS in the initial stage of NAFLD, it is also considered a promotor of HCC [125]. In fact, activation of Nrf2 was found to contribute to the progression of the preneoplastic lesion to malignancy, which was confirmed by in vivo detection of the inhibition of the Nrf2 pathway that accompanied the regression of cytokeratin 19-positive [125]. In addition, Nrf2 was found to take part in the protection processes of HCC cells by facilitating the survival response of FGF19 to endoplasmic reticulum stress [126].

One of the main hallmarks of cancer pathogenesis is metabolic reprogramming. In fact, HCC cells present a high DNL rate and survive in a highly rich lipid environment [127,128]. Thus, inhibition of lipogenesis might be a possible therapeutic target to counteract HCC progression. PGC-1β is the master regulator of oxidative metabolism, mitochondria biogenesis, and antioxidant response in the liver. However, in both genetic hepatic-specific PGC-1β-overexpressing (LivPGC-1β) and diethylnitrosamine (DEN)-induced HCC mouse models, PGC-1β plays an important role in HCC development, sustaining metabolic reprogramming through the induction of lipogenic enzymes and promoting tumor growth [128]. High levels of PGC-1β can boost reactive oxygen species (ROS) scavenger expression, therefore limiting the detrimental ROS accumulation and, consequently, apoptosis. Moreover, it supports tumor anabolism, enhancing gene expression in fatty acid and triglyceride synthesis. By contrast, the knockout of PGC-1β protects mice from developing HCC [128].

Mitochondrial dynamics have been also associated with cancer progression [129]. High metastatic HCC (MHCC-LM3, MHCC97-H, and MHCC97-L) cell lines appear to have more fragmented mitochondria due to the imbalance toward mitochondrial fission [130,131]. Z. Zhang et al. demonstrated that MFN1 overexpression, through the increase in mitochondrial fusion and expression of the epithelial marker E-cadherin, and reduction in expression of mesenchymal markers including N-cadherin inhibited tumor proliferation and subsequent metastatic potential [131]. MFN1 seems to also modulate metabolic reprogramming in HCC cells. Increased mitochondrial fusion by overexpression of MFN1 induced the expression of the main enzymes involved in OXPHOS and mediated metabolic shift from aerobic glycolysis to OXPHOS [131].

In summary, unbalanced mitochondrial dynamics alongside damaged mitochondria accumulation prompts metabolic reprogramming of hepatocytes, marked by the switch towards the Warburg effect, mutagenesis, epithelial–mesenchymal transition (EMT), and apoptosis escape, stimulating compensatory proliferation and HCC onset. Although mitochondrial metabolic reprogramming and mechanotransduction have been investigated in carcinogenesis, the crosstalk between the extracellular matrix and mitochondrial metabolism remains underexplored in NAFLD-driven HCC.

## 6. Conclusions

The prevalence of NAFLD is increasing at an impressive rate, and it is becoming the most common liver disease worldwide. The increase in NAFLD prevalence has led to an urgent need to develop better diagnostic and therapeutic approaches. In liver hepatocytes, multiple adaptive mechanisms are triggered to reduce liver fat accumulation and reestablish homeostasis. The failure of the adaptive mechanisms leads to OxS, ER stress, mitochondrial dysfunction, and inflammation, contributing to NAFLD progression. Indeed, mitochondria play a critical role in the advancement of NAFLD to NASH by decreasing mitochondrial FAO and continuing ATP depletion due to the loss of capacity to catabolize the excessive FFAs. During the development of NASH to HCC, the decreased FAO contributes to cancer cells adapting to the lipid-rich environment, and mitochondrial damage can also promote the activation of proliferative pathways. As mitochondrial dysfunction is considered a driving force in NAFLD progression, targeting these intracellular organelles and/or the pathways that can modulate their structure, function, and dynamics could contribute to establishing an effective therapy for NAFLD. However, a clear understanding of disease pathophysiology in its different stages is crucial but still not fully understood.

## 7. Future Perspectives

Regulation of different aspects of hepatocytes’ mitochondrial physiology decisively impacts the development and progression of NAFLD/NASH. Nevertheless, studies on distinct mitochondrial sub-population roles in NAFLD are still scarce, and their role in metabolic dysregulation remains poorly understood. Moreover, the impact of mitochondrial (mis)communication with other subcellular organelles (peroxisomes, lipid droplets, etc.) is unclear in NAFLD. Additionally, the impact of negligible pathways, such as fatty acid oxidation, DNL, and PPP, in the hepatocytes’ mitochondrial physiology in different stages of NAFLD is a topic to explore. As mitochondria are involved in the different stages of the disease, several molecular markers involved in mitochondrial metabolism, dynamics, and quality control mechanisms change differently across the disease spectrum; therefore, the study of these alterations may facilitate diagnosis. Finally, the impact of hepatocytes’ mitochondrial physiology in NAFLD-associated HCC remains unclear. Thus, there is a well-justified need to improve our understanding of different aspects of hepatocytes’ mitochondrial physiology in the context of NAFLD.

## Figures and Tables

**Table 1:** Expression patterns and function of genes associated with NAFLD risk.

Gene	Function	Variant	Outcomes in NAFLD
***PNPLA3***	Lipid remodeling; Lipogenesis	rs738409	Decreased lipolysis, phospholipase and retinyl-plamitate lipase activity; [26] Increased hepatic fat content, elevated liver enzymes, hepatic fibrosis, and cirrhosis [27]
***GCKR***	Glucose uptake; Lipogenesis	rs1260326	Inhibition of glucokinase; [28] Increased glycolytic flux and malonyl-CoA levels; [29] Increased hepatic fat storage and decreased β-oxidation [30]
***TM6SF2***	VLDL secretion	rs58542926	Increased hepatic TG content and higher risk of advanced fibrosis in NAFLD; [31] Lower concentration of hepatic-derived TG-rich lipoproteins; [32] Impaired incorporation of polyunsaturated fatty acids into hepatic TGs, phospholipids, and cholesterol ester [20]
***HSD17B13***	Lipid droplet remodeling; Retinol metabolism	rs72613567	Decreased risk of chronic liver damage in NAFLD patients; [33] Increased hepatic phospholipids and downregulation of inflammation-related genes [21]
***MBOAT7***	Remodeling of PI	rs641738	Increased liver damage; [24] Decreased PI species with arachidonoyl side chains; [23] Increased PI species with monounsaturated fatty acids; [23] Elevated plasma levels of LPI [24]

## Data Availability

Not applicable.

## Abbreviation

**8-OHdG** 8-Hydroxy-20-deoxyguanosine; **ACOX1** Peroxisomal acyl-coenzyme A oxidase 1; **ALCAT1** Acyl-CoA:lysocardiolipin acyltransferase-1; **ALT** Alanine transaminase; **AMP** Adenosine monophosphate; **AMPK** 5′ Adenosine monophosphate-activated protein kinase; **AST** Aspartate transaminase; **ATF6** Transcription factor 6; **ATG7** Autophagy-related protein 3; **ATG5** Autophagy-related protein 5; **ATG7** Autophagy-related protein 7; **BAT** Brown adipose tissue; **BMI** Body mass index; **BNIP3** BCL2/adenovirus E1B 19 kDa protein-interacting protein 3; **CAT** Catalase; **CHOP** C/EBP homologous protein; **CL** Cardiolipin; **CM** Cytosolic mitochondria; **CPT-1** Carnitine palmitoyltransferase-1; **CR** Caloric restriction; **DALYs** Disability-adjusted life years; **DAMPs** Danger-associated molecular patterns; **DEN** Diethylnitrosamine; **DGAT2** Diacylglycerol o-acyltransferase 2; **DNL** de novo lipogenesis; **ECM** Extracellular matrix; **ER** Endoplasmic reticulum; **ETC** Electron transport chain; **FAO** Mitochondrial fatty acid β-oxidation; **FFA** Free fatty acids; **GCKR** Glucokinase regulator; **GPAT1** Glycerol phosphate acyltransferase 1; **GPx** Glutathione peroxidase; **GSH** Glutathione; **H_2_O_2_** Hydrogen peroxide; **HCC** Hepatocellular carcinoma; **HDL-C** High-density lipoprotein cholesterol; **HFD** High-fat diet; **HMOX1** Heme oxygenase 1; **HSC** Hepatic stellate cells; **HSD17B13** hydroxysteroid 17-beta dehydrogenase 13; **IL-1β** Interleukin-1 beta; **IL-6** Interleukin-6; **IL-8** Interleukin-8; **INSR** Insulin receptor; **IR** Insulin resistance; **LC3** Microtubule-associated protein 1A/1B-light chain 3; **LDL-C** Low-density lipoprotein cholesterol; **LDs** lipid droplets; **LPI** Lysophosphatidylinositol; **MAFLD** Metabolic (dysfunction)-associated fatty liver disease; **MAM** Mitochondria-associated membranes; **MAPKs** Mitogen-activated protein kinases; **MBOAT7** Membrane-bound O-acyltransferase domain-containing 7; **MCD Methionine- and choline-deficient**; **MFN** Mitofusin; **MPC1** Mitochondrial pyruvate carrier 1; **mPTP** Mitochondrial permeability transition pore; **MST1** Macrophage stimulating 1; **mtROS** mitochondrial reactive oxygen species; **NADH** Nicotinamide adenine dinucleotide reduced form; **NAF** Non-alcoholic fibrosis; **NAFL** Non-alcoholic fatty liver; **NAFLD** Non-alcoholic fatty liver disease; **NASH** Non-alcoholic steatohepatitis; **NEFA** Nonesterified fatty acid; **NF-κB** Nuclear factor k-light-chain-enhancer of activated B cells; **NLRP3** NOD-like receptor family pyrin domain-containing 3; **Nrf2, NFE2L2** Nuclear factor erythroid-derived 2-like 2; **OPA1** Optic athrophy protein 1; **OXPHOS** Oxidative phosphorylation; **OxS** Oxidative stress; **PDM** Peridroplet mitochondria; **PGC-1** Peroxisome proliferator-activated receptor-gamma coactivator; **PI** Phosphatidylinositol; **PLIN5** Protein perilipin 5; ***PNPLA3*** Patatin-like phospholipase domain-containing 3; **PP2A** Protein phosphatase 2; **PPAR-α** Proliferator-activated receptor-α; **PPP** Pentose phosphate pathway; **PRDX6** Peroxiredoxin 6; **PTP1B** Protein-tyrosine phosphatase 1B; **ROS** Reactive oxygen species; **Sirt1** Sirtuin 1; **sn-1,2-DAGs** *sn*-1,2-diacylglycerols; **SOD** Superoxide dismutase; **SREBP1** Sterol regulatory element-binding protein 1; **StARD1** Steroidogenic acute regulatory protein; **T2DM** Type 2 diabetes mellitus; **TCA** Tricarboxylic acid; **TFAM** Mitochondrial transcription factor A; **TG** Triglycerides; **TLRs** Toll-like receptors; **TM6SF2** Transmembrane 6 superfamily member 2; **TNF-α** Tumor necrosis factor α; **UCP2** Uncoupling protein-2; **UPR** Unfolded protein response; **VLDL** Very low-density lipoprotein; **WHO** World Health Organization; **XBP1** Xbox-binding protein.
